# A Preliminary Investigation of the Interactions between the Brood Parasite *Chalcoela iphitalis* and Its Polistine Wasp Hosts

**DOI:** 10.3390/insects8030089

**Published:** 2017-08-24

**Authors:** Scott Nacko, Gregg Henderson

**Affiliations:** Entomology Department, Louisiana State University Agricultural Center, Baton Rouge, LA 70803, USA; grhenderson@agcenter.lsu.edu

**Keywords:** *Polistes*, *Mischocyttarus*, social, ectoparasite

## Abstract

The life history of *Chalcoela iphitalis*—a common brood parasite of social wasps—has been described in previous literature, but critical information regarding oviposition behavior and possible differential host parasitism remain cryptic. Here we report on infestation levels of this moth in field populations of paper wasps in *Polistes* and *Mischocyttarus*, as well as the oviposition behavior of the moths under a laboratory setting. We found evidence for differential parasitism between paper wasp genera in the field, with almost 50% nest infestation in *P. bellicosus* and no occurrences of moth infestation in *M. mexicanus*. Laboratory results revealed that oviposition occurs only at night and is stimulated by contact with the wasp nest or adult wasps. In this setting, eggs were laid largely on the substrate above or adjacent to the host nest, but not on the nest itself.

## 1. Introduction

The nests of eusocial paper wasps often support a diverse array of insect parasites and symbionts [1]. Understanding these insects with which paper wasps have co-evolved can ultimately help us understand the paper wasp ecology. The sooty-winged Chalcoela moth, *Chalcoela iphitalis* (Walker), is a brood parasite in the family Crambidae [2] which attacks Polistine wasps, including at least ten species of *Polistes* and one species of *Mischocyttarus* in the US and Central America [3]. However, evidence from a ten-year study indicated that *C. iphitalis* did not show a host preference between *P. fuscatus* and *P. dominula* [4]. Moth larvae feed on pupal or pre-pupal wasps before spinning silken cocoons containing layers of air pockets within wasp brood cells. Moth silk is not chewed through or removed by the wasps, and newly laid eggs and early instar wasp larvae can sometimes be observed on top of moth silk [3]. *C. iphitalis* overwinter inside the abandoned wasp nest and are typically bivoltine, with an adult emergence in the spring as well as late summer when nests containing brood are present. Infestations can be commonly observed, with up to 73% of nests parasitized in a population of one wasp species [5]. Parasitism of a population of *Polistes exclamans* in Texas by *C. iphitalis* has been detailed by Strassmann [5] over the course of three years. The average number of mature cells infested per nest ranged from 19–34% and varied significantly between years. Infestations usually peaked around July or August, and *C. iphitalis* may avoid ovipositing in previously infested nests. Each successful *C. iphitalis* larva kills one wasp pupa, but holes between cells were observed, indicating that some moth larvae may be feeding on multiple pupae.

The ability of *C. iphitalis* to destroy a large percentage of wasp pupae in a small period of time may have a large impact on worker replacement at the nest. Strassmann and Thomas [6] conducted a principal component analysis on a population of *P. exclamans* and found that nest decline was correlated with *C. iphitalis* infestation. A similar study concluded that heavy infestations of *C. iphitalis* can be a primary cause of colony failure. Moth diapause inside of old nests may be a cause for the rarity of nest re-use by paper wasps [7].

Paper wasp nests attach to a variety of substrates that include natural vegetation and manmade structures, and nest location is thought to be an important aspect related to moth invasion [1]. In one study, approximately 60% of the *Polistes* nests located on manmade structures in Illinois were parasitized by *C. iphitalis*, whereas only 20% of nests built on trees or shrubs were parasitized, suggesting that nests in vegetation may be less preferred or difficult for moths to locate [8]. 

Strassmann [5] notes that this moth lays eggs in wasp nests at night. Strassmann [5] and West-Eberhard [9] also described the parasite alarm reaction of adult wasps when encountering a moth, during which wasps will react violently by biting or stinging the area where the moth had been and subsequently alarming other wasps through vibrations and wing flipping on the nest. Wasps continuing the alarm behavior will sometimes leave the nest and walk over the substrate for up to 10 h after initial detection of the moth [5]. Mechanisms by which the moth parasite is able to bypass the host defense is still unknown.

The objective of this study was to determine parasitism rates in wasp species found in the Baton Rouge, Louisiana area, factors related to the parasitism, as well as oviposition behavior in the laboratory. To achieve this, we examined the behavior of both host and parasite in the laboratory, as well as the occurrence of *C. iphitalis* in field populations of Polistine wasps in southern Louisiana.

## 2. Materials and Methods

### 2.1. Field Study

Southern Louisiana study sites encompassed areas in Baton Rouge and St. Gabriel including Bluebonnet swamp, Burden Research Station, LSU Reproductive Biology Center, and LSU main campus. Areas were systematically searched for Polistine nests throughout the spring and summer of 2016. Because air temperature is a major factor affecting wasp activity [10], searches were carried out mostly on warm and sunny days when wasps were active, with search increments of 3–4 h each day. Areas for visual search included sheltered sites such as palm fronds, picnic shelters, eaves of buildings, as well as low shrub vegetation. Observations of wasps flying directly into a location sometimes led to the discovery of a nest in that manner. Upon discovery, each colony was identified to species and visited weekly for the duration of the season through colony decline. Presence of *C. iphitalis* infestation as well as percentage of cells infested were recorded. We used total cell number of nests that reached the pupal stage to calculate the percentage of cells with webbing (infested) for each species. Only nests that reached the pupal stage were included in our calculations due to the fact that *C. iphitalis* feeds only on pupal or pre-pupal wasp stages [3].

### 2.2. Laboratory Study

In July of 2016, two moth-infested wasp nests (*P. fuscatus* and *P. bellicosus*) which had been abandoned by the wasps were collected in the field. The nests were brought back to the laboratory and placed together in the bottom of a 12.7 × 10.16 cm cylindrical clear plastic container, whereupon 29 *C. iphitalis* adults eclosed within two days. Two active *Polistes dorsalis* nests without evidence of previous *C. iphitalis* infestation were also collected from the field and glued to the top of identical separate plastic containers as noted above; however, these containers were inverted such that the lid served as the bottom of the cage. Both nests were of similar size, and one nest (A) consisted of two adult females while the other nest (B) consisted of four adult females. All brood stages (eggs, larvae, and pupae) were present in both nests, which were maintained in the laboratory and given access to honey, water, and wax worms daily under a natural photoperiod. The two moth-infested wasp nests and two active *P. dorsalis* nests were collected at Burden Research Station in Baton Rouge, LA, USA.

To release moths from their cage to one of the two active *P. dorsalis* nests, container lids were removed and containers were placed together. This allowed the 29 moths in the lower cage to fly freely to the upper cage housing an active *P. dorsalis* nest for an immediate observation of 30 min. Two daytime (temperature = 23.1 °C and luminosity = 59) and two night-time (temperature = 21.9 °C and luminosity = 0) observations (28 and 29 July, data obtained from Onset HOBO^®^ UA-002 Pendant Temperature Light Data Logger) were conducted on each wasp nest. After the first 30-min daytime observation concluded, any moths remaining in the wasp cage area were placed back into their own cage, which was then placed underneath the next wasp nest for a 30-min observation. The behavior and interactions between wasp and moth were recorded. Because red light is not visible to most insects but provides light for observation by humans [11], a red light was utilized during night-time trials. After both 30-min night-time observations terminated, cages were separated and seven adult moths (from the 29 total) were placed in each wasp cage. Moths were then allowed further oviposition for 10 additional hours before being removed the next morning. This created a standard number of moths and oviposition time in each wasp cage. Removal of adult moths from wasp cages was facilitated by physical stimulation from a small paintbrush which encouraged downward flight into the moth cage. After each 10.5 h period, number of moth eggs and egg locations (wasp nest surface or cage surface including top, wall, or bottom) were noted in each wasp cage. In total, moths were permitted to oviposit in each wasp cage for 22 h (including daytime and night-time observational periods). Fourteen days after trials ended, two late instar wasp larvae were removed from each nest for examination of moth parasitism. Moth larval silk was noted in cells as evidence.

A two-way ANOVA procedure (SAS^®^9.4, ©2016 Proc Mixed, SAS Institute Inc., Cary, NC, USA) was utilized to analyze differences between moth egg positioning (either top, wall, or bottom of cage) and egg number in both wasp cages.

## 3. Results

### 3.1. Occurrence in Field Populations

We found 34 nests with evidence of parasitism out of 179 total nests which reached the pupal stage (an infestation rate of 18.9%). Infestations became noticeable (when moth larvae spun cocoons) as early as 1 June, with subsequent infestations noted throughout the summer until 6 October, whereupon no new infestations were observed. The species with the highest infestation was *Polistes bellicosus*, with 19 out of 46 (41%) of nests reaching the pupal stage becoming infested. *P. fuscatus* had 6 out of 28 (21%) infested, *P. dorsalis* had 6 out of 41 (14.6%), *P. metricus* had 2 out of 14 (14.2%), and *P. exclamans* had 1 out of 13 (7.6%) (Table 1). *Mischocyttarus mexicanus* had no observed cases of infestation out of 37 total nests which reached the pupal stage. On average, *P. metricus* (n = 2) had 79.4% cells infested, *P. bellicosus* (n = 19) 44%, *P. fuscatus* (n = 6) 33.4%, *P. dorsalis* (n = 6) 22.7%, and *P. exclamans* (n = 1) 19.7%. Average cells infested per nest for all species combined was 40%. Substrates that nests were built on which later became infested included Palmetto leaf (n = 17), wood (n = 8), painted wood (n = 5), metal (n = 2), plastic (n = 1), and cement (n = 1). No nests built on twigs or branches (n = 13) were infested by *C. iphitalis* (Table 2).

### 3.2. Polistes dorsalis Behavior under Laboratory Conditions

Moths quickly encountered adult wasps at the nest (n = 9 observations, five in nest A and four in nest B) during night-time trials only, whereupon some wasps would display parasite alarm behavior with jerking movements and wing flipping [3]. The degree to which parasite alarm behavior was displayed varied greatly among individual wasps; some females left the nest in pursuit of the moth or continued this behavioral pattern for the remainder of the 30-min observational period (n = 3), while other individuals spent very little or no time (n = 5) performing the same behaviors after contact with moths. One wasp in nest A left the nest in pursuit of contacting moths three times, spending ≈40 s before returning to the nest each time; no other wasps left their nest during trials. In the first nest (A) containing two adults, one female exhibited a much higher degree of alarm than the other. The less-alarmed female raised her wings often during contact with the more-alarmed female, but spent most of her time walking slowly around the nest. She was also observed grooming and cell checking during this time. The more alarmed female walked jerkily around the nest, flipped her wings, and was not observed grooming or cell checking for the full 30-min trials. Abdominal wagging was observed once on the face of the nest by the more alarmed female. In the second nest (B), one out of four females displayed the alarm behavior as previously described, however the behavior was displayed only for 20 min after initial contact with the moth before the wasp returned to resting behavior. Two of the remaining three females were recently eclosed (<24 h) and reacted to the alarmed female by moving away from her and toward the top of the nest. The fourth female raised her wings and made antennal contact with the alarmed female but did not exhibit the same degree of alarm.

### 3.3. Chalcoela iphitalis Behavior under Laboratory Conditions

**Figure 1:**
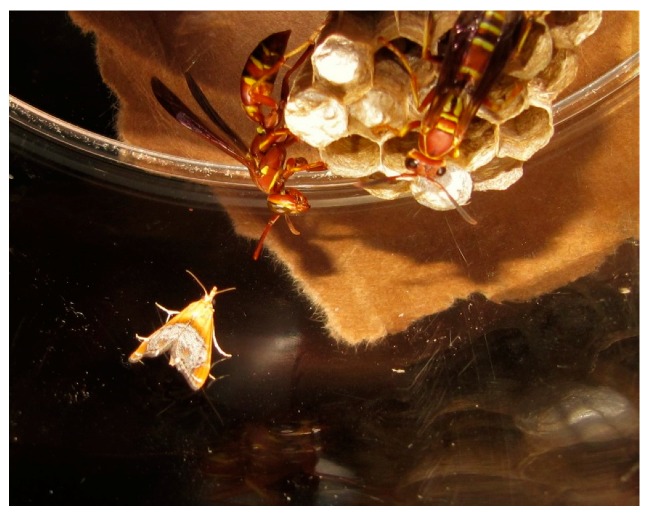
*Chalcoela iphitalis* approaches a *Polistes dorsalis* nest with antennae extended.

No wasp–moth interactions or moth flights were observed during daytime trials. Moths remained motionless on the walls and floor of their cage with antennae tucked back. During night-time trials, moths typically flew and landed within eight centimeters of the wasp nest with antennae fully extended and oscillating (Figure 1). 

**Figure 2:**
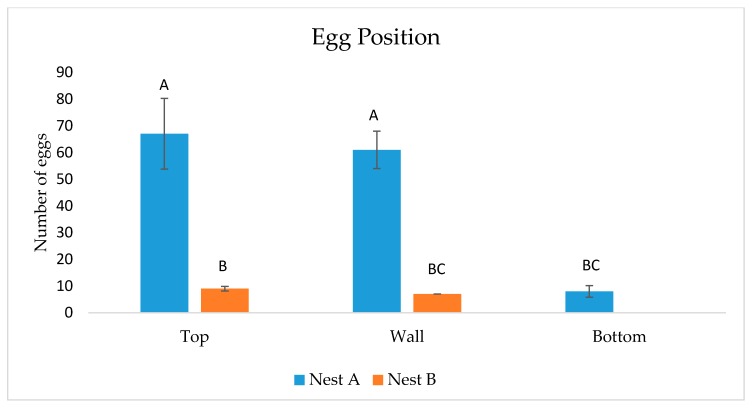
Number of *C. iphitalis* eggs found on various cage surfaces after 21 h. Different letters above bars represent significant differences.

**Figure 3:**
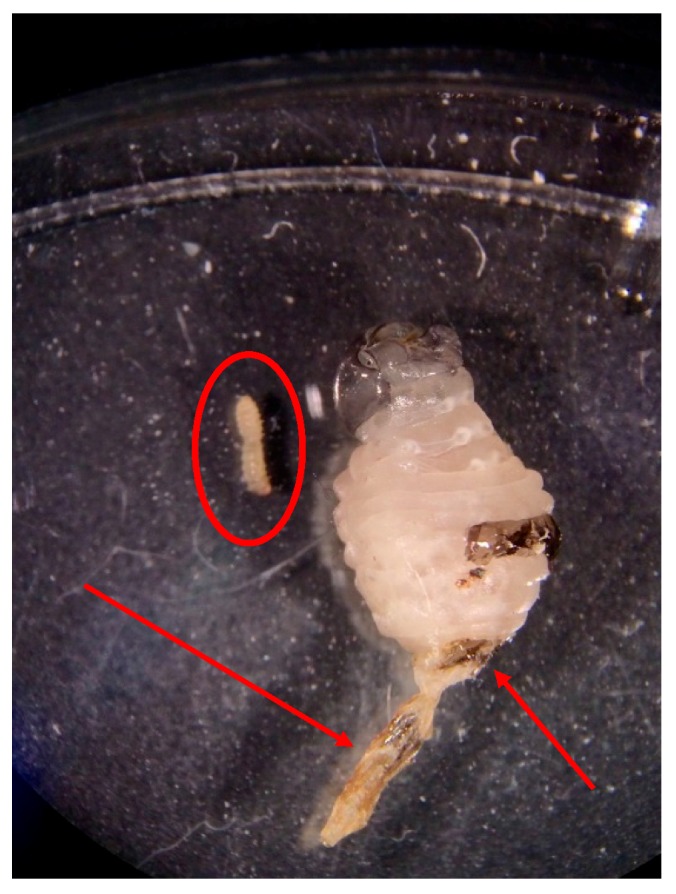
A live late instar *P. dorsalis* larva with arrows showing damage from feeding by *C. iphitalis* larva (circled).

In total, 14 moth–wasp or moth–nest contacts were observed. Moths and wasps made antennal contact during nine of these incidents, six of which resulted in moth oviposition within seconds of the encounter on the walls, ceiling, and floor of the cage, but never on the nest itself. When moths made antennal contact with an adult wasp, avoidance behavior was displayed as the moth tucked antennae back and ran rapidly in the opposite direction. Eggs were laid singly; often when an ovipositing moth would contact an existing egg on the cage surface with the tip of her abdomen, another egg would be deposited against it. This resulted in egg clumps of seven or less. Ovipositing females could be seen moving the tip of their abdomen from side to side as they walked along the surface of the cage. In two instances, moths hovered near the nest and made contact with the nest itself or an adult wasp before landing nearby and beginning oviposition. Moths were rarely (n = 1) observed walking on a nest surface. After completion of trials, one wasp cage (A) contained 136 *C. iphitalis* eggs, with 55 after the first 10.5-h period and 81 after the second, while the other cage (B) contained 16 eggs, 10 after the first period and 6 after the second. There was a significant difference between position of eggs in the cages (F_(2,12)_ = 10.84, *p* < 0.002), with the majority of eggs located on the walls and ceiling closest to the nest and few or no eggs on the bottom (Figure 2). Fourteen days after trials ended, all wasp pupae on each nest had eclosed, leaving only a few late instar larvae for us to examine. No evidence of moth silk was present by this time. Upon removal of a late instar larva from nest A, two *C. iphitalis* larvae (each ~4 mm in length) were found in the bottom of that cell, and the wasp larva showed signs of predation due to feeding, including a sclerotized, discolored, and deformed posterior end (Figure 3). Removal of a late-instar wasp larva from nest B revealed two larger (~7 mm) *C. iphitalis* larvae which moved into an adjacent cell through a hole in the cell wall. The adjacent wasp larvae showed no visible sign of predation. Moth silk was first observed in the nests four weeks after trials started, indicating a 4-week developmental time for *C. iphitalis* in the laboratory at a temperature of 22 °C.

## 4. Discussion

Although *Chalcoela iphitalis* attacks many different paper wasp species [3], our data suggest that the moth attacks *Polistes bellicosus* most often in this area, especially as compared with *Mischocyttarus mexicanus*. The only available host record for *C. iphitalis* from the genus *Mischocyttarus* is in *M. basimacula*, a species occurring in Central America [3]. In our study, *Polistes bellicosus* was the most commonly observed species and had the highest *C. iphitalis* infestation rate, while *Mischocyttarus mexicanus* was second most commonly observed species and had no infestations. This lack of infested *M. mexicanus* nests is of great interest, and several possibilities exist: enhanced behavioral and chemical defenses against the moth, insufficient host quality, more concealed nesting locations [8], or morphology of the two proximal abdominal segments, which are more elongate and stalk-like in *Mischocyttarus* than in *Polistes*. We speculate that this elongate petiole may add to the body flexibility for use of Van der Vecht’s gland—a sternal gland which secretes repellant compounds that protect the nest against ants, yellowjackets, and house flies [12]. Van der Vecht’s gland secretions have yet to be tested for repellency against parasitic Lepidoptera. It is plausible that the allomone produced by this gland in *Mischocyttarus*—which is typically applied to the nest petiole—prevents moth larvae from traveling down the petiole and into the comb in the same manner in which it prevents ants from doing so [13].

*C. iphitalis* has been noted to have no known host specificity within the *Polistes* genus [1]; however, a closely related species (*C. pegasalis*) with a similar life history to that of *C. iphitalis* was shown to have differential parasitism rates among *Polistes* species in Jamaica [14]. We found that *Polistes* species in our study were not attacked equally as often, and the percentage of infested cells also differed between species. However, the most frequently attacked species did not have the highest percentage of infested cells. This discrepancy may be due to the variation in the average number of cells built by each species [15]. If moths lay similar quantities of eggs at each nest, nests with fewer cells should have a higher percentage of total cells infested, which was indeed the case with *P. metricus*. A tradeoff between parasite vigilance behavior and foraging or brood care by adult female wasps was suggested as a possible explanation to differential parasitism among *Polistes* species [14]. However, oviposition of *Chalcoela* moths occurs mainly at night when wasps are not foraging [5], suggesting that other possible factors are at play. In the laboratory, we found that individual adult wasps exhibited a varying degree of alarm behavior after the detection of an adult moth. The wasp exhibiting the highest degree of alarm was also preforming abdominal wagging—a behavior observed more often in queens than in workers [16]. Avoidance of an alarmed individual by callow individuals in another instance was also observed, suggesting that alarm behavior may be correlated with social rank of the individual, as has been shown with aggression behavior [17].

Laboratory experiments also demonstrated that *Chalcoela iphitalis* oviposit on substrates surrounding the host nest rather than on the nest itself. This makes the substrate which the nest is built upon an important factor in avoiding predation by *C. iphitalis*. A substrate which has a large surface area (such as *S. palmetto* leaves or manmade structures) may be more conducive for moth oviposition, while a narrower substrate (such as twigs or small tree branches) may inhibit oviposition by the moth. In this study, we saw no infestation of nests built on twigs or branches in the field, supporting similar observations made by Reed and Vinson [8]. Antennal contact by a female moth with either an adult wasp or the wasp nest itself appeared to be a prerequisite for oviposition in the laboratory, but moths avoided walking onto or next to the nest. Antennal contact most likely provides host checking and oviposition stimulation, while avoidance behavior serves a function of remaining at a safe distance from defending wasps while ovipositing. Although the ectoparasitic moth larvae typically attack only pupae and prepupae [5], last instar wasp larvae were attacked in the laboratory. This could be due to the confined setting and lack of available pupae once all wasp pupae had matured. Field observations supported the basis of an exclusion of pre-pupal nests from the study, in that infestation of a pre-pupal nest was not observed. It is presumable that upon hatching, caterpillars located the nest pedicel and traveled into the nest, although this was not directly observed. Neonates likely go undetected by adult wasps due to their small size. Our results provide the first report of oviposition behavior of *C. iphitalis* and suggest differential host parasitism in field populations. Future studies focusing on behavioral differences of individual wasps during episodes of parasite invasion may provide the basis for differential alarm behavior among individuals within the colony, and provide further explanation for the differential parasitism by *Chalcoela* moths.

## Figures and Tables

**Table 1:** Average number of cells infested per species by *C. iphitalis* in field colonies of Polistine wasps. Based on nests containing brood in the pupal stage.

	*Polistes bellicosus* n = 46	*Polistes dorsalis* n = 41	*Polistes fuscatus* n = 28	*Polistes metricus* n = 14	*Polistes exclamans* n = 13	*Mischocyttarus mexicanus* n = 37
Nests with Infestation	19 (46%)	6 (14.6%)	6 (21%)	2 (14.2%)	1 (7.6%)	0
Total Cells	1061	472	486	25	203	0
Total Cells Infested	419 (39%)	96 (20%)	91 (18.7%)	18 (72%)	40 (19.7%)	0
Average Cells Infested per Nest	23 (44%)	16 (23%)	15 (33.4%)	9 (79.4%)	40 (19.7%)	0

**Table 2:** Number of nests and nests infested with *C. iphitalis* per substrate in field colonies of Polistine species.

Substrate	Number of Nests	Number of Infested Nests
Palmetto Leaf	120	17
Painted Wood	53	5
Metal	35	2
Un-painted Wood	22	8
Tree-Shrub	15	0
Cement	9	1
Plaster	6	0
Plastic	4	1
